# Structure of α-conotoxin BuIA: influences of disulfide connectivity on structural dynamics

**DOI:** 10.1186/1472-6807-7-28

**Published:** 2007-04-20

**Authors:** Ai-Hua Jin, Hemma Brandstaetter, Simon T Nevin, Chia Chia Tan, Richard J Clark, David J Adams, Paul F Alewood, David J Craik, Norelle L Daly

**Affiliations:** 1 Institute for Molecular Bioscience, Australian Research Council Special Research Centre for Functional and Applied Genomics, The University of Queensland, Brisbane QLD 4072, Australia; 2 School of Biomedical Sciences, The University of Queensland, Brisbane QLD 4072, Australia

## Abstract

**Background:**

α-Conotoxins have exciting therapeutic potential based on their high selectivity and affinity for nicotinic acetylcholine receptors. The spacing between the cysteine residues in α-conotoxins is variable, leading to the classification of sub-families. BuIA is the only α-conotoxin containing a 4/4 cysteine spacing and thus it is of significant interest to examine the structure of this conotoxin.

**Results:**

In the current study we show the native globular disulfide connectivity of BuIA displays multiple conformations in solution whereas the non-native ribbon isomer has a single well-defined conformation. Despite having multiple conformations in solution the globular form of BuIA displays activity at the nicotinic acetylcholine receptor, contrasting with the lack of activity of the structurally well-defined ribbon isomer.

**Conclusion:**

These findings are opposite to the general trends observed for α-conotoxins where the native isomers have well-defined structures and the ribbon isomers are generally disordered. This study thus highlights the influence of the disulfide connectivity of BuIA on the dynamics of the three-dimensional structure.

## Background

Nicotinic acetylcholine receptors (nAChRs) play a key role in the central and peripheral nervous system and are involved in neuronal growth and plasticity, development, learning, memory and pain sensation [1-5]. α-Conotoxins, found in the venoms of marine molluscs belonging to the genus *Conus*, act as antagonists at nAChRs and consequently have a range of potential therapeutic applications [6-8].

**Figure 1:**
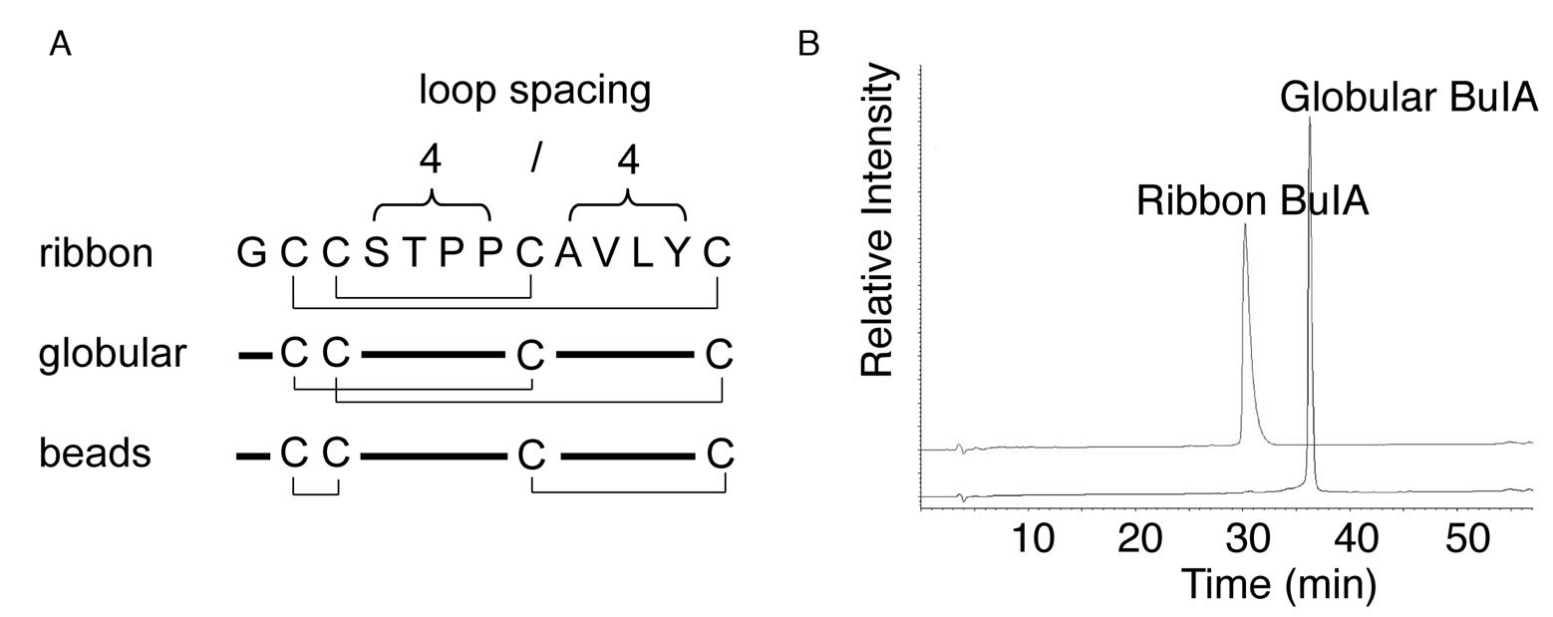
Disulfide isomers of BuIA. (A) A schematic representation of the ribbon, globular and beads disulfide connectivities of BuIA, a conotoxin having the unique 4/4 loop spacing. (B) HPLC traces of ribbon and globular BuIA. Analytical HPLC was carried out using a C_18 _Phenomenex analytical column (250 × 4.6 mm) with 90% acetonitrile and 0.043% trifluroacetic acid as the eluting solvent B and 1% B per minute as the gradient. Ribbon BuIA elutes earlier than the globular form but has a slightly broadened peak under the conditions utilized.

Members of the α-conotoxin family inhibit either muscle, neuronal or both types of nAChRs and prevent channel opening [6,9,10]. They comprise 12 to 19 amino acids, including four highly conserved cysteine residues that form two disulfide bonds. Native α-conotoxins have a disulfide connectivity linking the first and the third, and the second and the fourth cysteine residues (Cys^I^–Cys^III ^and Cys^II^–Cys^IV^), which is referred to as the "globular" isomer. The alternative connectivities (Cys^I^–Cys^IV ^and Cys^II^–Cys^III^) and (Cys^I^–Cys^II ^and Cys^III^–Cys^IV^) are referred to as the ribbon and beads forms, respectively and are shown in Fig. 1. The beads form has so far not been reported for naturally occurring conotoxins, but the ribbon connectivity is characteristic of the χ-conotoxins [6,11,12]. The segments between cysteine residues define two loops in the peptide backbone, also shown in Fig. 1.

The spacing between the cysteine residues in α-conotoxins is variable, leading to their classification into sub-families [6]. Table 1 shows the sequences of selected α-conotoxins with different cysteine spacings. The original α-conotoxins discovered in the 1980's, such as the paralytic toxin GI, have a 3/5 spacing, with three amino acids in the first and five in the second loop [13]. They compete mainly for nAChRs of the muscle endplate. Members of the "non-classical" α-conotoxins display 4/X spacings (where X is 3, 4, 6, or 7) and target mainly neuronal nAChRs, with the exception of EI, which is muscle specific [14,15]. In addition to the variations in cysteine spacing there is a significant degree of sequence variation and it is this diversity that results in the exquisite selectivity of the α-conotoxins. The subtype selectivity of selected α-conotoxins is illustrated in Table 1.

BuIA is the only α-conotoxin with the unique 4/4 spacing (see Table 1 and Fig. 1) and is the first conotoxin identified in the piscivorous *Conus bullatus *species. Azam *et al. *[16] used conserved features of the α-conotoxin signal sequence and 3'-untranslated sequence region of α-conotoxin prepropeptide genes to clone the gene coding for BuIA. A synthetic version of the corresponding 13-amino acid mature peptide with the globular disulfide connectivity (Cys^2^–Cys^8^, Cys^3^–Cys^13^) is active against α3- and α6-containing nAChR subtypes, whereas α2- and α4-containing nAChRs are blocked less effectively. Strikingly, BuIA kinetically discriminates between β2- and β4-containing receptors, as the off-rates are rapid for β2-subunit, but very slow for β4-containing nAChRs. This feature appears to extend across different mammalian species, as similar results for mouse, rat and human nAChRs were obtained [16]. Thus the novel α-conotoxin BuIA appears to be a valuable probe to distinguish among nAChRs containing different α and β subunits.

Understanding the structure-activity relationships of α-conotoxins has been a major focus of conotoxin research in recent years and, as a result the structures of many of the known α-conotoxins have been determined. Conotoxins are particularly suitable for structural analysis with NMR spectroscopy because of their small size, solubility and generally well-defined conformations, and the majority of α-conotoxin structures have been determined using this technique [17,18]. Despite the range of sequences and spacings between the cysteine residues a well-defined consensus structural motif has been found for the α-conotoxins, with the major features being the restraints imposed by the conserved globular disulfide connectivity and a helical region centred around Cys^II ^[18]. In the current study we have examined the structures and activities of the globular isomer and the non-native ribbon isomer of BuIA with a Cys^2^–Cys^13^, Cys^3^–Cys^8 ^connectivity and show that the globular isomer is structurally heterogeneous while the non-native ribbon isomer has a well-defined conformation.

## Results

Orthogonal syntheses of globular and ribbon BuIA were achieved using solid phase peptide synthesis via Boc chemistry. Purification via HPLC yielded a single product for each isomer whose mass was determined using electrospray ionization mass spectrometry. Both isomers have an observed mass of 1311.6 Da (calculated mass 1311.5 Da). HPLC traces for the purified products are shown in Fig. 1B, from which it is clear that the ribbon form elutes earlier than the globular form, indicating that its hydrophobic residues are buried to a greater extent. Interestingly, under the HPLC conditions used the ribbon form has a slightly broader peak than the globular form despite having a more defined structure as described below. When prepared without selective protection of the cysteine residues BuIA folds predominately into the globular isomer in a 4:2:1 ratio (globular:ribbon:beads), consistent with this being the native form in venom, as is the case with all other α-conotoxins. For the oxidation profile of BuIA without selective protection see Additional file 1.

**Figure 2:**
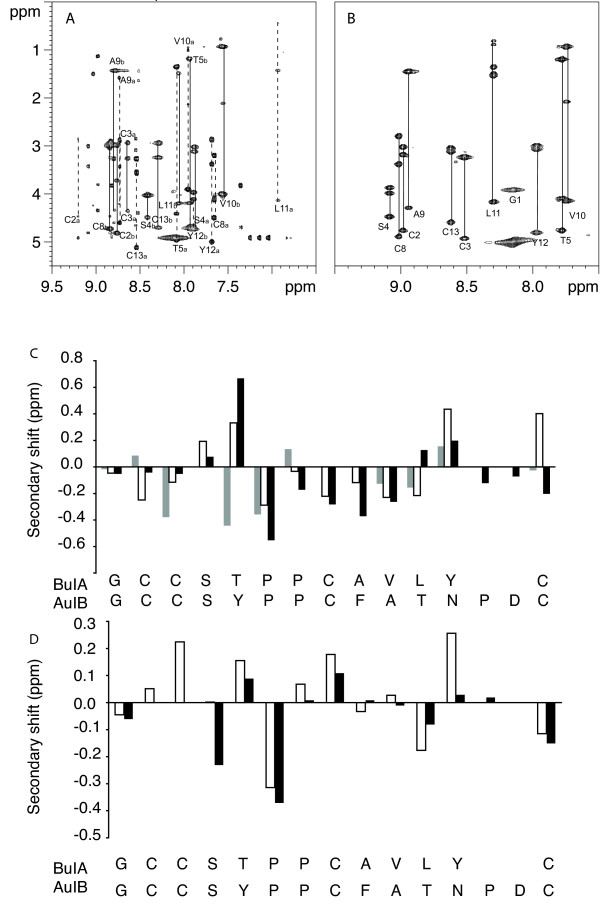
NMR analysis of ribbon and globular BuIA. The upper panels show the amide regions of the TOCSY spectra at 600 MHz of globular (A) and ribbon BuIA (B) at 282 K in 95% H_2_O/5% ^2^H_2_O at pH 3. The one letter code and the residue numbers are used for labeling. For globular BuIA (A) stretches of assigned residues are labeled, as a or b to indicate conformers A and B. The lower panels (C & D) show a secondary shift analysis of conformers A and B from globular BuIA and ribbon BuIA compared to AuIB. For each residue, represented by the one letter code, the differences between the actual chemical shifts for the α-protons and their respective random coil shifts are graphed. (C) Native AuIB (black bars), conformer A (white bars), conformer B (grey bars). (D) Ribbon AuIB (black bars), ribbon BuIA (white bars).

The globular and ribbon isomers purified from oxidation without selective protection were analyzed by NMR spectroscopy and found to be identical to their respective isomers isolated following selective formation of the disulfide bonds. However, the TOCSY and NOESY spectra revealed considerable differences between the two isomers, as illustrated in the upper panels of Fig. 2. The non-native ribbon form with the Cys^2^–Cys^13^, Cys^3^–Cys^8 ^disulfide connectivity displayed spectra consistent with a well-defined structure with a single conformation. Specifically, it yielded spectra with good dispersion of chemical shifts in the amide region ranging from 7.7 to 9.1 ppm and exactly the expected number of TOCSY spin systems. In contrast, the spectra of globular BuIA displayed more than the expected number of TOCSY cross-peaks, indicating the likely presence of conformational heterogeneity. The possibility that the extra cross-peaks were due to impurities was eliminated because only a single sharp HPLC peak was observed and only a single mass was detected by mass spectrometry.

The sequential assignment of the spectra of the ribbon and globular forms was undertaken using a combination of TOCSY and NOESY spectra [19]. For the ribbon isomer the assignment was straightforward. By contrast, the presence of multiple sets of peaks due to three conformers (referred to here as conformers A, B and C), a lack of detection of sufficient sequential NOESY cross-peaks and peak overlap prevented complete sequential assignment of all of the globular BuIA conformers. Nonetheless, with the aid of the 900 MHz spectra conformers A and B were assigned, as indicated in Fig 2A. The first six residues of conformer C were assigned but the proline residue at position 6 has weak spectral peaks and has an αH shift overlapped with several other residues and thus assignment of Pro7 and sequentially following residues was not possible. A range of pH values (3, 4 and 5.5), temperatures 280–310 K and acetonitrile co-solvent concentrations (20% or 40% v/v) were used to determine if solution conditions influenced the presence of multiple conformations but similar ratios of isomers were present in all the conditions used.

Conformers A and C both display sharp peaks, in contrast to conformer B, which has significant broadening. Based on peak integrals the ratio of the isomers is approximately 3:2:1 (A:B:C). Because peaks from conformers A and C in the same solution are sharp, the broadened peaks of conformer B presumably do not reflect aggregation, but are likely to be the result of exchange broadening. Thus, not only does the globular isomer lead to at least three conformers in slow exchange on the NMR time scale, but one set of peaks probably reflects the presence of two or more conformers in intermediate to fast exchange. Overall this isomer of BuIA appears to be much more conformationally heterogeneous than other conotoxins studied to date.

Inspection of the sequential cross-peaks associated with the proline residues indicated that the conformational heterogeneity observed for globular BuIA originates from *cis-trans *isomerisation of the two proline residues. Conformer A has both proline residues in a *trans *conformation, consistent with the report of Chi *et al. *[20] whereas conformer B has a *trans *Pro^6 ^and *cis *Pro^7 ^based on strong NOEs observed between the α- and δ-protons of Thr^5 ^and Pro^6 ^and a strong αH-αH NOE between Pro^6 ^and Pro^7 ^respectively. The lack of a complete assignment for conformer C and peak overlap prevented determination of the proline configurations for this conformation. In the ribbon form of BuIA, which adopts an ordered structure, the same proline conformation pattern as conformer B was observed, namely *trans *Pro^6 ^and *cis *Pro^7^.

Analysis of the secondary αH chemical shifts of ribbon BuIA indicates a lack of regular secondary structure. There is no continuous stretch of residues exhibiting negative deviations that would be indicative of helices or positive deviations indicative of extended structures, such as β-sheets. Panel D of Fig. 2 shows a comparison of the αH secondary shifts of ribbon BuIA with the ribbon isomer of AuIB [21]. The trends are similar, but in general the secondary shifts of ribbon BuIA deviate from random coil to a greater extent than that observed for ribbon AuIB consistent with a well-defined structure for ribbon BuIA.

Despite the lack of complete assignment of all the conformations of globular BuIA an analysis of the chemical shifts was very informative. The secondary shifts of conformer A are very similar to the related α-conotoxin AuIB [21] as shown in Fig. 2C, indicating that the native conotoxin fold is present [18]. In contrast, the secondary shifts of conformer B differ significantly from those of AuIB (Fig. 2D). While our work was in progress the structure of the native conformation (i.e. conformer A) of BuIA was reported by Chi *et al *and confirms that the consensus structure of the α-conotoxins is maintained [20]. It was not possible to directly compare our findings with those of Chi *et al *because chemical shifts or spectra were not provided in the study but consistent with our study, Chi *et al *reported the presence of additional peaks that they presumed arose from a *cis *conformation in the globular isomer. In the present study, we have shown there is more than one additional conformation present. The ribbon isomer, which is the focus of our study, was not examined in the Chi *et al *study [20].

Given the single conformation observed for ribbon BuIA it was of interest to determine the three dimensional structure of this isomer. Structure calculations were carried out with 96 NOE distance restraints (comprising 12 long-range, 18 medium-range, 50 sequential and 16 intraresidual distance restraints) and six dihedral angle restraints, using a simulated annealing protocol in CNS. The ϕ-angle restraints, derived from αN coupling constants, were -65 ± 15° for Ala^9 ^and Leu^11^, -120 ± 15° for Thr^5^, and -120° ± 30° for Cys^8^. The ϕ-angle for Cys^13 ^was restrained to 100 ± 80° based on the intraresidual Hα-HN NOE being clearly weaker than the NOE between HN and the Hα of the preceding residue. The χ_1_-angle of Cys^8 ^was restrained to 180 ± 30° based on the αβ coupling constants and NOE intensities. Hydrogen bonds were not included in the calculations but measurement of the exchange rates for the amide protons of ribbon BuIA revealed that 3 of the 11 amides, namely Val^10^, Leu^11 ^and Tyr^12^, are slowly exchanging. Their amide proton signals were still detected in NMR spectra recorded 30 minutes after dissolution of the sample in ^2^H_2_O, but all amide protons had exchanged after 45 minutes in ^2^H_2_O. This result suggests that Val^10^, Leu^11 ^and Tyr^12 ^are more protected from the solvent than are the other residues.

**Figure 3:**
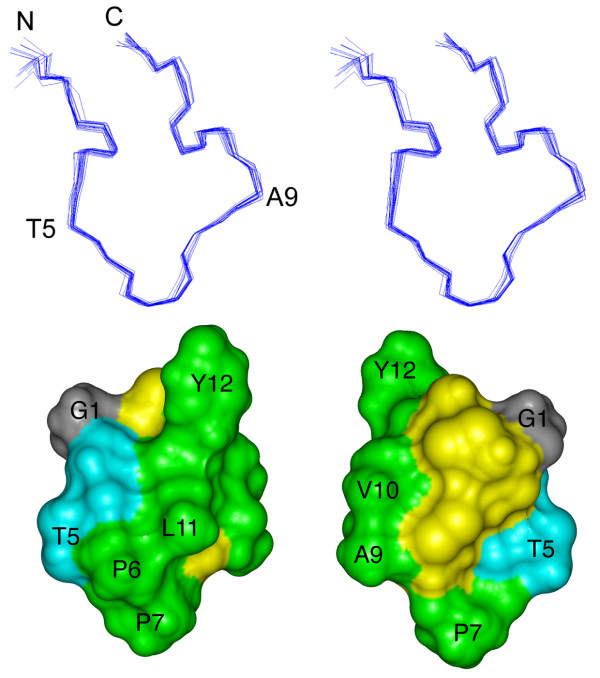
Three-dimensional structure of the ribbon isomer of BuIA. (top) Stereoview of the 20 NMR-derived lowest energy structures of ribbon BuIA. For clarity only the backbone (N, C^α^, C') is shown and the two disulfide bonds are omitted. The structures are superimposed over the whole molecule. N refers to the N-terminus; C refers to the C-terminus. Residues were labeled with the one letter code. (bottom) Solvent accessible surface of ribbon BuIA. The two views are rotated by 180° about the vertical axis. Hydrophobic residues (Pro, Ala, Val, Leu, Tyr) are green, hydrophilic residues (Ser, Thr) are blue, Gly is grey and the Cys residues are yellow. Selected residues are labeled with the one letter code.

Fig. 3 shows a stereoview of the superposition of the 20 lowest energy structures of ribbon BuIA. Despite its small size it adopts a well-defined three-dimensional structure. The ensemble of structures aligns well over the whole molecule with a pairwise backbone RMSD of 0.36 ± 0.12 Å and a RMSD of 1.1 ± 0.49 Å for all heavy atoms, and had good structural and energetic statistics, as shown in Table 2. No NOE violations higher than 0.3 Å and no dihedral angle restraint violations greater than 3° were observed within these 20 structures. In the Ramachandran plot, showing the favored ϕ- and ψ-angle combinations for residues in proteins, all residues of ribbon BuIA (except Pro and Gly) lie in the most favored and additionally allowed regions.

Using the program PROMOTIF [22] the 20 lowest energy structures of ribbon BuIA were examined to identify consensus structural elements. In agreement with the prediction from the secondary shifts, ribbon BuIA shows no α-helices or β-sheets. The structure comprises an inverse γ-turn for residues 2–4 but no regular secondary structure is present in the remainder of the molecule. One of the two disulfide bonds stabilizing the three dimensional structure, namely that formed by Cys^3 ^and Cys^8^, overlays very well in the 20 lowest energy structures. In 17 of 20 structures the torsion angles of this disulfide bond are representative of a right-handed hook [23]. By contrast, the disulfide bond joining Cys^2 ^to Cys^13 ^is classified as right-handed hook in 9 out of 20 structures but not recognized as a standard disulfide type in the other structures. Calculation of the hydrogen bonds using MolMol [24] revealed that in 13 of 20 structures the amide proton of Leu^11 ^is bonded to the carbonyl of Ala^9^. This hydrogen bond is consistent with the relatively slow exchange of the amide proton of Leu^11^, but Val^10 ^and Tyr^12 ^were also slowly exchanging and no hydrogen bond acceptors were apparent from the calculated structures.

Using the program InsightII the solvent accessible surface of ribbon BuIA was calculated and is shown in Fig. 3, from which the large hydrophobic surface formed by Ala, Val, Leu, Tyr and Pro residues is clear. The hydrophobic patch extends across a wide stretch of the whole molecule, as it comprises only a few hydrophilic residues, namely Ser and Thr. Strikingly, there are no charged residues in the molecule. Tyr^12 ^protrudes conspicuously, making the overall shape of ribbon BuIA non-spherical. In contrast to many other α-conotoxins the cysteine residues are rather exposed to the solvent, presumably as a result of the ribbon disulfide connectivity and the fact that BuIA is only 13 residues in length.

**Figure 4:**
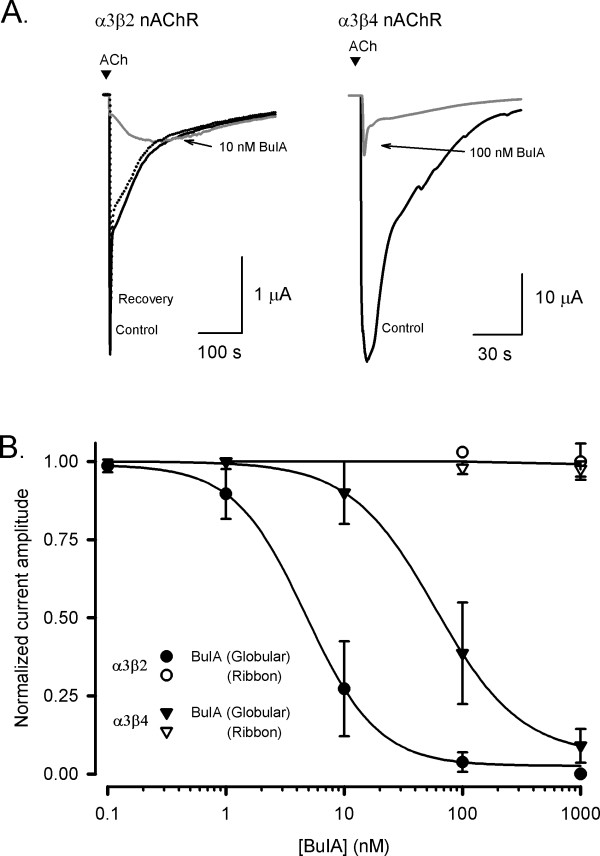
Effect of globular and ribbon isomers of α-conotoxin BuIA at nAChR subtypes expressed in *Xenopus *oocytes. (A)Representative ACh-evoked currents mediated by α3β2 and α3β4 nAChR subtypes obtained in the absence (control) and presence of 10 nM and 100 nM BuIA-globular, respectively. Complete recovery was observed after 10 min washout (broken line). (B)Concentration-response curves for the inhibition of α3β2 and α3β4 nAChRs by globular (filled symbols) and ribbon (open symbols) isomers of BuIA. Best fit of the data gave IC_50 _values of 4.8 ± 0.4 nM (*n*_H _= 1.3) and 59.1 ± 2.3 nM (*n*_H _= 1.2) for α3β2 and α3β4 nAChRs, respectively, whereas the ribbon isomer of BuIA exhibited no inhibition at 1 μM. Responses are shown as a percentage of ACh (100 μM)-induced peak current amplitude after a 5 min incubation of the BuIA isomers with respect to control (ACh alone). Error bars are SEM with n = 4–7 for each data point.

The biological activities of the BuIA globular and ribbon analogues, as well as a form where the cysteine residues were alkylated, were examined in an electrophysiological assay by measuring the % inhibition of ACh-evoked current amplitude at the α3β2 and α3β4 nAChR subtypes expressed in *Xenopus *oocytes (Fig. 4A). The alkylated form of BuIA was analyzed by mass spectrometry and had an observed molecular mass (1571.9 Da) consistent with the calculated mass (1571.64 Da). Membrane currents were evoked with 100 μM ACh and concentration-response curves were fitted to determine IC_50 _values and Hill coefficients. The concentration-response curves for inhibition of α3β2 and α3β4 nAChR subtypes by globular BuIA gave IC_50 _values of 4.8 ± 0.4 nM (*n*_H _= 1.3) and 59.1 ± 2.3 nM (*n*_H _= 1.2), respectively, whereas almost no inhibition was observed with 1 μM ribbon BuIA at either nAChR subtype (n = 4–7; Fig. 4B). Furthermore, the globular BuIA isomer is approximately ten-fold more potent at β2- compared to β4-subunit containing nAChR subtypes. These results for synthetic globular BuIA are consistent with that reported previously [16]. Alkylated BuIA was inactive at α3β2 and α3β4 subtypes when tested at 10 nM and 100 nM respectively. Increasing the concentration of alkylated BuIA to 10 μM also failed to inhibit nAChRs.

## Discussion

As BuIA is the only α-conotoxin reported so far with a 4/4 spacing its three dimensional structure is of significant interest. In the current study we examined the structural features and determined the biological activity of the globular (Cys^I^–Cys^III^, Cys^II^–Cys^IV^) and ribbon (Cys^I^–Cys^IV^, Cys^II^–Cys^III^) disulfide isomers of BuIA. These studies show that the general trend observed for α-conotoxins where the native globular form has a well-defined conformation and the ribbon isomer is disordered is reversed for BuIA.

NMR analysis of the globular isomer of BuIA revealed more cross-peaks than expected for a 13 residue peptide. At least three conformations are present in the two-dimensional spectra of globular BuIA and as native α-conotoxin structures determined to date generally display well-defined structures, the significant heterogeneity differs from usual findings for α-conotoxins. Surprisingly, this conformational heterogeneity is not seen in the ribbon isomer, which forms a single well-defined conformation. The observation that the HPLC trace of the ribbon reveals a broad peak, but the structure is well-defined is interesting and unusual. One possibly is that the different conditions under which the HPLC and NMR are carried out are responsible for these differences. The more hydrophobic environment used for the HPLC analysis coupled with the presence of TFA could potentially affect how the ribbon isomer behaves on RP-HPLC. However, recording NMR spectra of globular BuIA in the presence of varying amounts of acetonitrile co-solvent did not influence the conformational heterogeneity, making the sharper peak observed by HPLC analysis for globular BuIA compared to ribbon BuIA a puzzling result.

**Figure 5:**
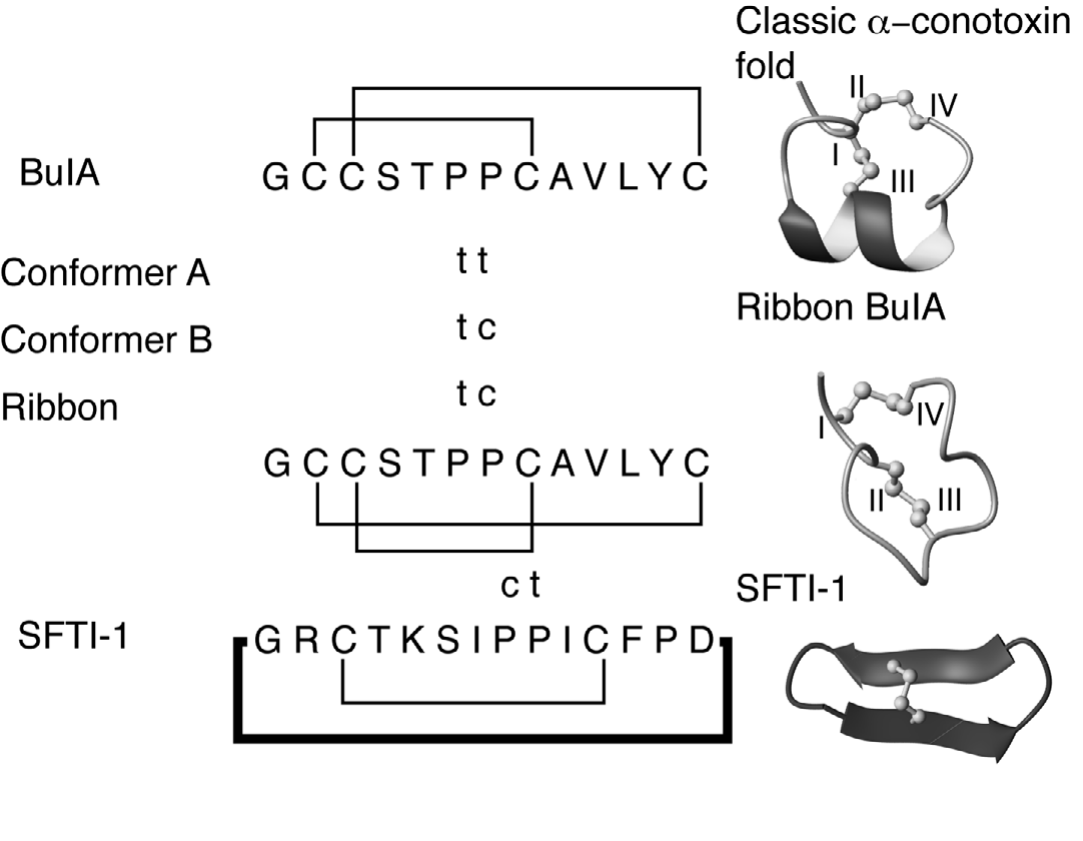
Proline geometries of the BuIA isomers and the sunflower trypsin inhibitor SFTI-1 [26]. The proline residues with *trans *geometry are marked as t and those with *cis *as c. The three-dimensional structures of a classic α-conotoxin, ribbon BuIA and SFTI-1 are shown to highlight the structural differences between the various peptides.

The conformational heterogeneity of globular BuIA originates form *cis/trans *isomerisation of the pair of adjacent proline residues in the middle of the sequence. The proline configurations for the globular isomers and ribbon BuIA are highlighted in Fig. 5. Conformer A has both proline residues in a *trans *conformation, consistent with the trend observed for other native α-conotoxins, whereas conformer B is broadened and displays a *trans/cis *configuration for Pro^6 ^and Pro^7 ^respectively. The conformations of the proline residues in conformer C were not determined because of the lack of a complete assignment. Ribbon BuIA displays the same proline geometry as conformer B despite the difference in disulfide connectivity. The BuIA sequence can clearly accommodate a range of geometries of the proline residues in contrast to what has generally been observed in previous structural analyses of α-conotoxins [17,18]. One exception is α-conotoxin GI that has previously been shown to have two interconverting conformations [25] but this differs from BuIA where there are clearly multiple conformations. Other peptides are known to contain similar proline motifs to BuIA, including SFTI-1[26], and in that case the Pro-Pro motif has a *cis/trans *configuration as shown in Fig. 5. This highlights the complexity that can be associated with the geometry of proline residues in small cysteine-rich peptides and how changes in structure and sequence can result in different geometries being favored.

The structurally heterogeneous globular isomer of BuIA is active on α3-containing neuronal nAChRs, with a preference for receptors comprising β2- rather than β4-subunits. This specificity is quite similar to some of the 4/7 α-conotoxins such as MII [20]. The structure of conformer A of globular BuIA, determined by Chi *et al *[20], has been compared to that of MII and it appears that the two-turn helical motif present in both MII and BuIA is important for binding to the α3-subunit of neuronal nAChRs [20]. Recent studies on the binding of α-conotoxins to the acetylcholine binding protein, a homolog of the nAChR extracellular domain, have shown that the rigid consensus structure found in solution does not change significantly upon binding [27-29]. Thus, as ribbon BuIA does not contain the consensus structural motif it consequently exhibits no inhibitory activity at nAChRs up to 1 μM. It appears that the well-defined conformation of ribbon BuIA prevents it from adopting an appropriate conformation for binding to the nAChR.

We have also shown here that an analogue of BuIA with the cysteine residues reduced and alkylated is inactive at the nAChR. This is consistent with studies on ImI where the reduced and alkylated form had a significant decrease in activity at the α7 receptor [30]. Given that the reduced and alkylated form of BuIA is likely to be devoid of structure based on the strong influence of disulfide bonds on the structures of small cysteine-rich peptides, the lack of activity of this peptide indicates that it cannot adopt an appropriate conformation for binding to the nAChR. Thus, the linear sequence alone is not sufficient for activity and a propensity to form a helical structure in solution appears to be correlated with activity at the nAChR.

**Figure 6:**
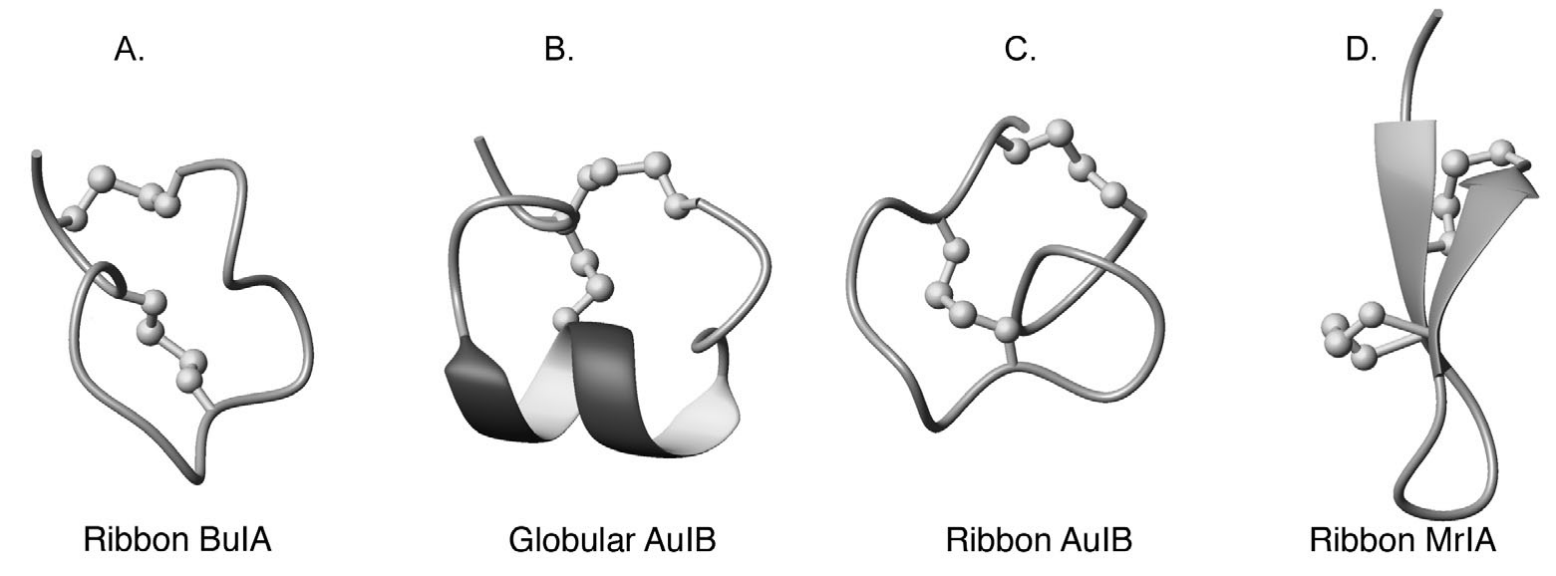
Backbone trace of the structures of ribbon BuIA (A), globular AuIB (B), ribbon AuIB (C), and ribbon MrIA (D). Disulfide bonds are shown as balls and sticks. The helices are shown with thickened ribbons and β-strands are indicated with arrows.

The ribbon isomer of BuIA, despite having a well-defined structure, is not the preferred isomer formed during air oxidation using standard conditions applicable to other conotoxins. The fact that the folding pathway favors the globular disulfide connectivity indicates that it is energetically more favorable, even though multiple conformations are present in solution. Although the ribbon isomer is less favoured during oxidation, as is often the case for non-native isomers, it displays significantly different properties to both non-native and native conotoxin ribbon isomers. The ribbon isomers of both α-AuIB and α-GI exhibit greater conformational flexibility than their globular counterparts [21,31], in contrast to ribbon BuIA, indicating that the unique sequence of BuIA stabilizes the ribbon isomer. Structures of globular and ribbon AuIB are shown in Fig. 6. Although a single structure is shown for the ribbon isomer of AuIB it displays considerable conformational heterogeneity relative to the globular isomer. Furthermore, the ribbon isomer of BuIA lacks any regular secondary structure, in contrast to the χ-conotoxins that naturally contain a ribbon connectivity. The χ-conotoxin MrIA consists of a β-hairpin connected by an inverse γ-turn [32], as shown in Fig. 6.

Another similarity between the ribbon isomers of α-conotoxins is related to the geometry of the proline residues [6,18]. The inactive ribbon isomers of BuIA and GI [31] both contain a *cis *proline (Pro^7 ^and Pro^5 ^respectively) in contrast to the *trans *Pro seen in the native disulfide connectivity for all α-conotoxin structures determined to date [18]. In one conformation of globular BuIA (conformer B, Fig. 2) Pro^7 ^is also in a *cis *geometry, but the alternative *trans *conformation may be stabilized upon binding to the receptor. The implications for the presence of the *cis *proline in activity have not yet been elucidated.

## Conclusion

The native globular isomer of BuIA, although being the favored isomer during oxidative refolding, has multiple conformations in solution, unlike the majority of native α-conotoxins. In addition, the structure of the ribbon isomer is stabilized whereas all other non-native isomers display disordered structures. Overall our results highlight the influence of the disulfide connectivity of BuIA on the dynamics of the three-dimensional structure. This information is potentially important for on-going efforts to understand the structure-activity relationships of this valuable class of peptides.

## Methods

### Materials

Protected N-α-Boc-L-amino acids and reagents used for peptide chain assembly were purchased from NovaBiochem. Methylbenzhydrylamine resin was from Peptide Institute Inc. (Osaka, Japan). *p*-Cresol and *p*-thiocresol were purchased from Aldrich (Sydney, Australia). HBTU was purchased form Richelieu Biotechnologies (Quebec, Canada). Anhydrous HF was from Matheson Gas (BOC Gases, Melbourne, Australia). All of the solvents used for HPLC and peptide synthesis were purchased from Labscan, (Bangkok, Thailand).

### Peptide synthesis

BuIA analogues were manually assembled using a stepwise *in situ *neutralization protocol for Boc chemistry [33]. The four Cys residues were differentially protected in pairs corresponding to the globular and ribbon isomers with HF-labile methylbenzyl groups and HF-resistant acetamidomethyl groups. An additional synthesis was also done without selective protection of the cysteine residues. Other amino acid side-chain protection was as follows: Tyr (BrZ), Thr (Bzl) and Ser (Bzl). All the syntheses were carried out on a 0.25 mmol scale using HBTU (0.5 M/DMF) as the activation reagent, and DIEA as neutralizing reagent on MBHA resin. Neat trifluoroacetic acid was used as the amine-deprotecting reagent. Deprotection and cleavage were carried out in HF:*p*-cresol:*p*-thiocresol (9:0.5:0.5) for 2 h. Methylbenzyl groups attached to cysteines were removed during the HF cleavage and the oxidation of the first disulfide bond was performed in 0.1 M NH_4_HCO_3 _oxidation buffer at pH 8 at a concentration of 0.1 mg/mL. The one pair disulfide bonded peptides were purified by HPLC and lyophilized prior to the formation of the second disulfide bond. Simultaneous deprotection and oxidation of acetamidomethyl protected cysteine residues was performed in 80% methanol at low pH. Iodine was added and the reaction mixture was stirred for 5 min under argon. Methanol and iodine were removed by rotary evaporation. The resultant mixture was diluted 10-fold and purified by RP-HPLC. The peptide synthesized without selective protection of the cysteine residues was oxidized in 0.1 M NH_4_HCO_3 _pH 8, and 1 mM reduced glutathione. Linear reduced BuIA was alkylated with iodoacetamide in 0.1 M ammonium acetate buffer for testing in biological assays.

### RP-HPLC and mass spectrometry

Preparative HPLC were performed on Waters 600E solvent delivery system. Data were collected via a 484-absorbance detector from Waters at 230 nm. Analytical HPLC was performed on Shimadzu LC-2010 instruments. Preparative HPLC was carried out using a Phenomenex C_18 _column (250 × 21.20 mm, 10 μm). Analytical HPLC was performed on a Phenomenex C_18 _(250 × 4.6 mm, 10 μm) column. 90% acetonitrile and 0.043% trifluoroacetic acid was used as the eluting solvent B. 0.05% trifluoroacetic acid was used as the eluting solvent A. 1%B per minute was used as the linear gradient at standard column flow rates (8 mL/min for preparative HPLC and 1 mL/min for analytical HPLC). Electrospray mass spectra were acquired on a Micromass LCT (Manchester, UK), a liquid chromatography-orthogonal acceleration reflecting TOF mass spectrometer, coupled to an Agilent HPLC system. Samples (~5 μL) were injected into a moving solvent (70 μL/min, 70% acetonitrile/formic acid 0.05% in water) coupled directly to the electrospray ionisation source. Full scan mass spectra were acquired over the mass range of 400–1600 Da with a scan step size of 0.2 Da. Molecular masses were derived from the observed m/z values.

### NMR spectroscopy

Although X-ray crystallography has been used in a few cases to solve conotoxin structures [34,35] NMR has been the predominant technique for structurally characterizing this class of peptides[17]. Samples for ^1^H NMR measurements contained ~1 mM peptide in 95% H_2_O/5% D_2_O (v/v) at pH 3, 4 and 5.5. Spectra on ribbon BuIA were recorded at 282 or 290 K on Bruker AVANCE-500, 600 and 900 spectrometers whereas a range of temperatures between 280 and 310 K were used for globular BuIA to determine if temperature influenced the multiple conformations present. 2D NMR spectra were recorded in phase-sensitive mode using time-proportional phase incrementation for quadrature detection in the *t*_1 _dimension. The 2D experiments consisted of a TOCSY using a MLEV-17 spin lock sequence with a mixing time of 80 ms, DQF-COSY, ECOSY and NOESY with mixing times of 100–250 ms. Solvent suppression was achieved using a modified WATERGATE sequence. Spectra were acquired over 6024 Hz with 4096 complex data points in F_2 _and 512 increments in the F_1 _dimension. Identification of slowly exchanging amide protons was achieved by acquisition of one-dimensional and TOCSY spectra immediately following dissolution of a fully protonated peptide in ^2^H_2_O·^3^J_HN-Hα _coupling constants were measured from a one-dimensional spectrum or from the DQF-COSY spectrum.

Spectra were processed on a Silicon Graphics Indigo workstation using XWINNMR (Bruker) software. The *t*_1 _dimension was zero-filled to 1024 real data points, and 90° phase-shifted sine bell window functions were applied prior to Fourier transformation. Chemical shifts were referenced to internal 2, 2-dimethyl-2-silapentane-5-sulfonate.

### Structure calculations

Preliminary structures of ribbon BuIA were calculated using a torsion angle simulated annealing protocol within the program DYANA [36]. Final structures were calculated using simulated annealing and energy minimization protocols within CNS version 1.1 [37]. The starting structures were generated using random (ϕ,ψ) dihedral angles and energy-minimized to produce structures with the correct local geometry. A set of 50 structures was generated by a torsion angle simulated annealing protocol [38]. This protocol involved a high-temperature phase comprising 4000 steps of 0.015 ps of torsion angle dynamics, a cooling phase with 4000 steps of 0.015 ps of torsion angle dynamics during which the temperature was lowered to 0 K, and finally an energy minimization phase comprising 500 steps of Powell minimization. Structures consistent with restraints were subjected to further molecular dynamics and energy minimization in a water shell, as described by Linge and Nilges [39]. The refinement in explicit water involved the following steps. First, heating to 500 K via steps of 100 K, each comprising 50 steps of 0.005 ps of Cartesian dynamics. Second, 2500 steps of 0.005 ps of Cartesian dynamics at 500 K before a cooling phase where the temperature was lowered in steps of 100 K, each comprising 2500 steps of 0.005 ps of Cartesian dynamics. Finally, the structures were minimized with 2000 steps of Powell minimization. Structures were analyzed using PROMOTIF [22] and PROCHECK-NMR [40].

### Electrophysiology

RNA was prepared from linearized cDNA encoding the rat α3,β2 and β4 nAChR subunits (provided by Dr. J. Patrick, Baylor College of Medicine, Houston, TX) and transcribed using a mMESSAGE mMACHINE™ high yield capped RNA transcription kit. *Xenopus *laevis were anesthetized with oocytes surgically removed and placed in OR2 buffer (82 mM NaCl, 2 mM KCl, 1 mM MgCl_2 _and 5 mM HEPES at pH 7.4) with 3 mg/ml collagenase (Sigma) for 1–2 hrs at room temperature then stored in ND96 buffer (96 mM NaCl, 2 mM KCl, 1.8 mM CaCl_2_, 1 mM MgCl_2 _and 5 mM HEPES at pH 7.4) supplemented with 50 mg/l gentamycin and 5 mM pyruvic acid. Oocytes were injected with 5 ng of cRNA and kept at 18°C in ND96 buffer for 2–5 days before recording.

Membrane currents were recorded from *Xenopus *oocytes using the two electrode (virtual ground circuit) voltage clamp technique with an automated workstation with eight channels in parallel, and an on-line analysis (OpusXpress™ 6000A workstation, Molecular Devices, Union City, CA). Both the voltage recording and current injecting electrodes were pulled from borosilicate glass (GC150T-15; Harvard Apparatus, Edenbridge, UK) and filled with 3 M KCl with resistances between 0.2 – 1.5 MΩ. All recordings were conducted at room temperature (20 – 23°C) using a bath solution of ND96 as described above. During recordings, the oocytes were perfused continuously at a rate of 1.5 ml/min. Acetylcholine (100 μM) was applied for 2 s at 5 ml/min with 10 min washout periods between successive applications with 5 min incubations of the BuIA analogues. Cells were voltage clamped at a holding potential of -80 mV. Data were sampled at 500 Hz and low pass filtered at 200 Hz. Peak current amplitude was measured before and after the incubation of BuIA analogues, using the empirical Hill equation to calculate the IC_50 _and Hill coefficient (*n*_H_) of the concentration-response curves. The data were fitted by a non-linear least squares algorithm (SPSS inc. Chicago, IL) with pooled data represented as mean ± SEM.

## Abbreviations

HPLC, high performance liquid chromatography; NMR, nuclear magnetic resonance; ACh, acetylcholine; nAChRs, nicotinic acetylcholine receptors.

## Authors' contributions

A-HJ designed experiments and carried out synthetic studies. HB carried out structural studies and helped draft the manuscript. STN designed and carried out biological assays. CCT carried out synthetic and folding studies. RJC designed and carried out synthetic and folding studies. DJA designed biological experiments and revised the manuscript. PFA conceived study and designed synthetic experiments. DJC designed structural studies and revised the manuscript. NLD designed and carried out structural studies and drafted and revised the manuscript. All authors have read and approved the final manuscript.

## Supplementary Material

Additional File 1 Oxidative folding of BuIA. The data provided represent the oxidation of conotoxin BuIA without selective protection of the cysteine residues. Click here for file

## Figures and Tables

**Table 1:** Sequences and activities of selected α-conotoxins

***Name***	***Spacing^a^***	***Sequence***	***Receptor^b^***	***Species***	***Ref.***
GI	3/5	E**CC**-NPA**C**GRHYSC*	M α1β1γδ	*C. geographus*	[13]
ImI	4/3	G**CC**SDPR**C**AWRC*	N α7	*C. imperialis*	[41]
BuIA	4/4	G**CC**STPP**C**AVLYC*	N α6/α3 β4/β2	*C. bullatus*	[16]
AuIB	4/6	G**CC**SYPP**C**FATNPDC*	N α3 β4	*C. aulicus*	[42]
PnIA	4/7	G**CC**SLPP**C**AANNPDYC*	N α3 β2	*C. pennaceus*	[43]
**Cys**	**I**	**II**	**III**	**IV**	

^*a *^number of residues between the second and third, and the third and fourth cysteines

^*b *^M, muscle nAChR; N, neuronal nAChR

* C terminal amide

Sequences, in one letter code, are aligned using the conserved cysteines forming disulfide bonds, shown in bold.

**Table 2:** NMR and refinement statistics for ribbon BuIA

**NMR distance & dihedral constraints**	
Distance constraints	
Total NOE	96
Sequential (|i-j| = 1)	50
Medium-range (|i-j| < 4)	18
Long-range (|i-j| > 5)	12
Intra-residual	16
Total dihedral angle restraints	
Phi	5
chi1	1
**Structure statistics**	
Violations (mean and s.d.)	
Distance constraints (Å)	0.05 ± 0.003
Dihedral angle constraints (°)	0.61 ± 0.2
Max. dihedral angle violation (°)	3
Max. distance constraint violation (Å)	0.3
Deviations from idealized geometry	
Bond length (Å)	0.004 ± 0.00025
Bond angles (°)	0.53 ± 0.02
Impropers (°)	0.35 ± 0.02
Average pairwise r.m.s.d.** (Å)	
Backbone	0.36 ± 0.12
Heavy atoms	1.1 ± 0.49
Ramachandran statistics (%)	
Most favoured	55.6
Additionally allowed	44.4
Disallowed	0.0

**Pairwise r.m.s.d. was calculated among 20 refined structures
