# Supplementary material for: Structure of α-conotoxin BuIA: influences of disulfide connectivity on structural dynamics
Source: BMC Struct Biol. 2007 Apr 20;7:28. doi: 10.1186/1472-6807-7-28 (PMC1865545; doi:10.1186/1472-6807-7-28)
Supplement: Additional File 1 — Oxidative folding of BuIA. The data provided represent the oxidation of conotoxin BuIA without selective protection of the cysteine residues. [file 1472-6807-7-28-S1.doc]

Supplementary information


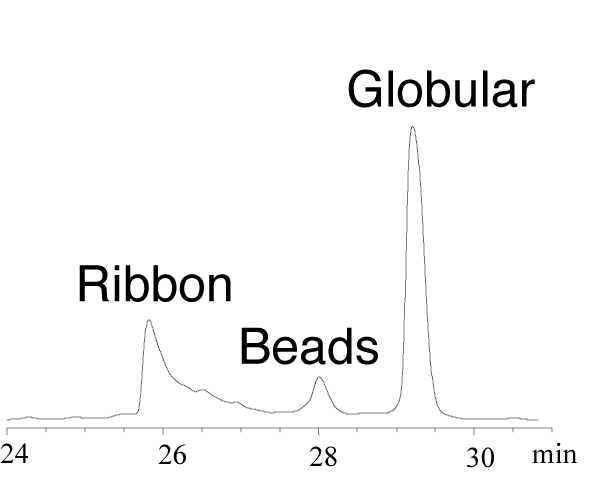


Oxidative refolding of α-BuIA Oxidation was performed in 0.1 M NH_4_HCO_3_ (pH 8.0) and 1mM reduced.
